# Methods for Real-Time Prediction of the Mode of Travel Using Smartphone-Based GPS and Accelerometer Data

**DOI:** 10.3390/s17092058

**Published:** 2017-09-08

**Authors:** Bryan D. Martin, Vittorio Addona, Julian Wolfson, Gediminas Adomavicius, Yingling Fan

**Affiliations:** 1 Department of Statistics, University of Washington, Box 354322, Seattle, WA 98195-4322, USA; 2 Department of Mathematics, Statistics, and Computer Science, Macalester College, St. Paul, MN 55105-1899, USA; addona@macalester.edu; 3 Division of Biostatistics, School of Public Health, University of Minnesota, Minneapolis, MN 55455-0341, USA; julianw@umn.edu; 4 Department of Information and Decision Sciences, Carlson School of Management, University of Minnesota, Minneapolis, MN 55455-0438, USA; gedas@umn.edu; 5 Humphrey School of Public Affairs, University of Minnesota, MN 55455-0395, USA; yingling@umn.edu

**Keywords:** mode prediction, movelets, dimension reduction, classification

## Abstract

We propose and compare combinations of several methods for classifying transportation activity data from smartphone GPS and accelerometer sensors. We have two main objectives. First, we aim to classify our data as accurately as possible. Second, we aim to reduce the dimensionality of the data as much as possible in order to reduce the computational burden of the classification. We combine dimension reduction and classification algorithms and compare them with a metric that balances accuracy and dimensionality. In doing so, we develop a classification algorithm that accurately classifies five different modes of transportation (i.e., walking, biking, car, bus and rail) while being computationally simple enough to run on a typical smartphone. Further, we use data that required no behavioral changes from the smartphone users to collect. Our best classification model uses the random forest algorithm to achieve 96.8% accuracy.

## 1. Introduction

Smartphones have become ubiquitous in the lives of billions. The development of smartphone technology has led to the incorporation of increasingly advanced sensors, opening up a number of opportunities for data mining applications. In particular, smartphones generate a constant stream of data describing the phone’s acceleration (via an onboard accelerometer) and location (via the Global Positioning System (GPS)). Among other possible tasks, these data streams can be used to predict a user’s mode of transportation (i.e., whether an individual is traveling by car, bus, bike, rail or on foot) in near real time.

Learning about a smartphone user’s mode of transportation has many useful applications. Government agencies currently collect transportation information using surveys. This requires time-consuming manual data collection, nearly one hour on average for a one-day survey for a single household [1], and is prone to response bias and inaccuracy [2]. GPS data are more representative of actual societal transportation patterns [3].

Smartphone applications could utilize the user’s mode of transportation to automatically adjust cellphone settings. For example, a user could create a driving play list that automatically starts when he/she begins driving a car or an exercise play list that automatically starts when he/she begins biking. A user could also receive automatic alerts, such as a notification to remind himself/herself about an oil change when he/she has driven a certain number of miles. The mode of transportation could also be of value to advertisers, including those advertising transportation-related services (e.g., bike shops) or entertainment (e.g., restaurants). Finally, consumers may be interested in learning more about their day-to-day lives. The user mode of transportation information could be stored and presented to users as an intuitive and quantified representation of individualized information. For example, users could see how much time they spent driving or how much distance they have walked, in a given period.

In this paper, we use smartphone GPS and accelerometer sensor data to classify a user’s mode of transportation. Data were collected to replicate real-world settings, meaning that smartphones were carried in a usual manner in order to replicate the type of data an actual smartphone application would receive. Further, we focus on methods that aim to simplify the data as much as possible while retaining predictive accuracy. This allows our methods to be utilized in a standard smartphone without substantial burden on battery life. We distinguish between five modes of transportation: walking, biking, car, bus and rail.

Some prior research has investigated mode of transportation detection using either accelerometer or GPS data. Bao and Intille [4], Krishnan et al. [5] and Kwapisz et al. [6] made predictions based on accelerometer data gathered under lab-controlled settings, using multiple accelerometers placed at various locations on the human body. Brezmes et al. [7] and Györbíró et al. [8] used accelerometer data from smartphones. Other work has focused on GPS data. In Patterson et al. [9], GPS data and bus route information are combined to classify between car, bus and foot. Sohn et al. [10] and Anderson and Muller [11] use Global System for Mobile Communication (GSM) data to distinguish between stationary, walking and driving. Zheng et al. [12] use GPS data to classify between bike, walk, bus and car. We extend this work to develop models that include both GPS and accelerometer data, as well as a focus on data that are not collected under lab-controlled conditions.

More recently, researchers have developed algorithms that combine both GPS and accelerometer data. Reddy et al. [13] combined these data to classify between motorized transportation, walking, running and biking, employing a decision tree followed by a first-order discrete hidden Markov model. Shafique and Hato [14] investigated dichotomous output, using binary logistic regression to compare the performance of classification when splitting the data by ranking, one against the rest and one against all. Shafique and Hato [15] compared the performance of support vector machines, adaptive boosting, decision trees and random forests. They distinguished between walk, bicycle, car and train and showed that it is optimal to classify modes of transportation using data in equal proportion. Feng and Timmermans [16] used a Bayesian belief network to compare predictions from only GPS data, only accelerometer data and the combination of the two. They found that accelerometer data work better than GPS alone, but achieved their best accuracy using the combined data. Xia et al. [17] also used GPS and accelerometer data to distinguish between walking, bicycling, stationary and motorized transport. They employ discrete fast Fourier transform and ant colony optimization to reduce the dimensions of the data. Our method contributes to this area by emphasizing a focus on dimension reduction and model simplicity, as well as our choices for the mode of transportation.

Some smartphone applications exist that aim to classify activities. Many focus on fitness, such as trying to estimate the length of a user’s run. Others, such as Anguita et al. [18], focus on smartphones used in a real-world manner to distinguish between activities. They distinguish between different movements, including walking, going upstairs, going downstairs, standing, sitting and laying. Google’s ActivityRecognitionApi [19] does not distinguish between motorized modes of transportation. The work of Ellis et al. [20] is similar to ours, but they do not distinguish between different modes of motorized transportation, and they use accelerometers and GPS devices placed at specific locations (e.g., on the hip and attached to wooden boards carried in a backpack) to ensure a uniformity that cannot be replicated in authentic settings. We could find no example of an application that focuses on addressing the computational burden of the classification of our modes of transportation in order to address issues of phone battery life using data that requires no behavioral changes to collect.

For this paper, we compared three techniques for predicting the mode of transportation from smartphone GPS and accelerometer data, in near-real time. The first technique is an extension of the “movelets” approach introduced by Bai et al. [21]. Movelets are a dictionary-based machine learning technique based on matching time series vectors. We extended the approach to handle the two parallel time series defined by period GPS and accelerometer measurements. The second and third techniques are specialized versions of *k*-nearest neighbors and random forests, with clustering and partitioning based on a set of movement features extracted from the GPS and accelerometer time series. Because the number of such features is potentially very large, we incorporated two dimension reduction strategies—principal components analysis (PCA) and recursive feature elimination (RFE)—into each of these feature-based techniques. We employed several levels of dimension reduction to balance our goals of model accuracy and model complexity. Our work makes three main contributions to the literature. First, we extended the movelets technique to accommodate bivariate time series. Second, we combined dimension reduction techniques with existing classification techniques to create more effective mode classification algorithms. Third, we compared the performance of our proposed techniques on data collected in a “real-world” setting.

Section 2 describes the source of our data, the preprocessing techniques used on the data and the feature generation process. Section 3 outlines the movelet classification algorithm. Section 4 explains the movelet, *k*-nearest neighbor and random forest techniques and also introduces the two dimension reduction methods (i.e., PCA and RFE). The performance of the three techniques is evaluated in Section 5. We provide some concluding remarks in Section 6.

## 2. Materials and Methods

The data were collected by six students participating in an undergraduate summer project on analyzing smartphone sensor data in the summer of 2014. There were two male and four female participants, all ranging in age from 18–25. The data were originally collected only for internal use by the students as part of their project, so IRB approval was not sought at the time of collection. When preparing this manuscript, the authors sought and obtained permission from all students participating in the summer project to use these data and to make them publicly available. The data consist of time-indexed speed and accelerometer readings along with mode types; no location information is kept after calculating speed, and the data are not individually identifiable.

The smartphones were carried in ordinary locations such as pockets and handbags during typical daily travel; notably, these data are not collected in a lab-controlled setting. The six individuals collected 96.59 h of GPS data and 98.62 h of accelerometer data (the minor discrepancy comes from instances when the GPS signal was lost). The GPS data were recorded once per second (a rate of 1 Hz), and the accelerometer data were recorded at a rate of five per second (5 Hz), resulting in 347,719 GPS observations and 1,775,137 accelerometer observations.

### 2.1. Preprocessing

Participants used an application provided on the smartphone to record the start and end times of trips. For each trip, they indicated the true mode of transportation: walking, biking, car, bus and rail. This will likely lead to some human error within the data, as it is impossible to change the mode setting at the exact moment in which the mode of transportation is actually changed. Additionally, mode transitions may yield intrinsic properties uncharacteristic of any of our five modes of interest. For example, the act of getting on a bus may not have properties that the phone will be able to identify as either bus or walking, nor were we interested in including such brief and uncharacteristic moments in our training data. We thus removed all data within 10 s (both before and after) of a mode change. Moreover, we removed all trips that did not contain at least 120 s of data. Trips that are too short may be uncharacteristic of the mode they represent. Such trips may also be too short to create the desired time series features for accurate classification. After preprocessing, we had 2.64, 6.91, 8.25, 7.30, and 21.62 h of bike, bus, car, rail and walking data, respectively.

From the GPS coordinates, we created a vector of speed values measured in miles per hour. Previous work (e.g., [9]) use GPS data to collect geocoded information such as bus routes. We intentionally do not include such information so that our algorithm requires only smartphone sensor data. This allows our algorithm to be more generalizable by removing the dependence on location-specific data that must be downloaded and is often subject to change. Smartphones come equipped with a tri-axial accelerometer; however, the axes on which acceleration is measured are fixed relative to the device itself and not relative to the Earth’s axes. In real-world settings, smartphones are not attached to the body in a fixed position, so the device orientation changes over time. While it is possible to calculate a stationary phone’s orientation relative to the Earth’s axis by applying an appropriate rotation matrix, these calculations become inaccurate for a phone in motion (since the rotation matrix changes over time), and hence, the three separate components of acceleration $\left(a_{x},a_{y},a_{z}\right)$ are difficult to interpret. Therefore, we computed the linear magnitude of the acceleration vector, $\left||a|\right|=\sqrt{a_{x}^{2}+a_{y}^{2}+a_{z}^{2}}$. For simplicity, in what follows, we refer to the linear magnitude ||a|| simply as “acceleration”.

### 2.2. Notation

We briefly introduce some notation that we will use in describing the methods in the next section. Let $f_{A}$ and $f_{S}$ represent the measurement frequency of speed and acceleration, respectively. In our case, $f_{A}$ and $f_{S}$ are 5 Hz and 1 Hz, respectively. Then, at time *t*, we define two stochastic processes:

$$\begin{array}{c} S_{<}\left(t\right)=\left\{S\left(t-1/f_{S}\right),S\left(t-2/f_{S}\right),\ldots \right\}\\ A_{<}\left(t\right)=\left\{A\left(t-1/f_{A}\right),A\left(t-2/f_{A}\right),\ldots \right\} \end{array}$$

where $S_{<}\left(t\right)$ represents the stochastic process for GPS (speed) data and $A_{<}\left(t\right)$ represents the stochastic process for accelerometer data. Let M(t) be the mode of transportation at time *t* and *T* be the total time interval over which the data are collected. Our goal is to develop an algorithm to predict M(t) using $S_{<}\left(t\right)$ and $A_{<}\left(t\right)$ for any t∈T.

## 3. Bivariate Movelets

Our first method is an extension of the so-called “movelets” technique described in [21] for predicting position changes (standing, sitting, lying down, etc.) on the basis of accelerometer readings. The method (which we will refer to as the univariate movelet approach) involves partitioning accelerometer time series data into segments (“movelets”) and clustering the segments known to be from the same mode (e.g., standing) to define a set of characteristic accelerometer time series “signatures” for that mode. For new data, the mode is predicted by determining which mode contains movelets that most closely match the observed time series. Since our data contain both speed and accelerometer time series data, we extended the ideas of Bai et al. to create bivariate movelets, as described below.

### 3.1. Generating Bivariate Movelets

Consider the *j*-th sequence of accelerometer and GPS measurements that are known to come from a single mode of transportation $M_{j}$ (e.g., car). Let $T_{j}$ be the length of time covered by the sequence (e.g., 20 min). We define a window length *H* to be the length of time covered by each movelet; in our application, we chose H=30,60,90 and 120 s. At a single time point $t\leq T_{j}$, we define the following two movelets consisting of values from the accelerometer and GPS time series:

$$\begin{array}{cc} A_{j}\left(t\right) & =\{A_{j}\left(t-1/f_{A}\right),A_{j}\left(t-2/f_{A}\right),\ldots ,A_{j}\left(t-H/f_{A}\right)\}\\ S_{j}\left(t\right) & =\{S_{j}\left(t-1/f_{S}\right),S_{j}\left(t-2/f_{S}\right),\ldots ,S_{j}\left(t-H/f_{S}\right)\} \end{array}$$

Let $X_{j}\left(t\right)=\left(A_{j}\left(t\right),S_{j}\left(t\right)\right)$, and let Δ be the length of time by which we slide the moving window across the interval $\left[0,T_{j}\right]$ to define the sequence of movelets. In our case, Δ is 5 s. This value was intentionally chosen to induce overlap in the movelets. Each time *t* is included in $\frac{H}{\Delta }$ movelets, with the exception of times near the beginning or end of the measurement in a given mode. As a result, the starting number of movelets is roughly equal to the number of seconds of data divided by five. By design, these movelets include overlap and redundancy, an issue that we address in Section 3.2 and Section 3.3. The full set of movelets on $\left[0,T_{j}\right]$ is given by $X_{j}=\left\{X_{j}\left(t\right),t=\Delta ,2\Delta ,\ldots ,T\right\}$. The full set of bivariate movelets for each mode *m* is collected together to form a “chapter”,

$$C_{m}=\underset{j:M_{j}=m}{⋃}X_{j}$$

In our setting, we formed five chapters corresponding to car, bus, bike, rail and walking. The pseudocode for generating a generic movelet chapter is described in Algorithm 1.
**Algorithm 1** Pseudocode for Generating a Movelet ChapterGiven data Z(t) of length *T* with known class *m*, sliding window Δ and length of movelet *H*Initialize empty chapter $C_{m}=\left\{\emptyset \right\}$, and i=0While (Δ×i)+H≤T:           $C_{m}=C_{m}\cup \left\{Z\left(\left(\Delta \times i\right)+1\right),\ldots ,Z\left(\left(\Delta \times i\right)+H\right)\right\}$           i=i+1

### 3.2. Validation of Training Movelets

Depending on the amount of available data for mode *m*, the chapter $C_{m}=\left\{\left(A_{m}\left(t\right),S_{m}\left(t\right)\right),t:M\left(t\right)=m\right\}$ may contain thousands of bivariate movelets. Many of these movelets may be redundant or otherwise unhelpful for predicting the mode of transportation for new data. Hence, we sought to identify the subset of movelets in $C_{m}$ that are most informative for classification purposes. After the movelets are created, they are partitioned into training, validation and testing sets with simple random sampling. We used 60% of the data for training, 20% for validation and 20% for testing. We calculated the Euclidean distance between each validation movelet and all training movelets in order to identify the “most useful” movelets (as described in Section 3.3) in the training data and to avoid storing duplicate information. The goal of doing this is to retain only the movelets that are most characteristic of their assigned mode of transportation, as well as to reduce the size of the dictionary. If a movelet is not close to at least some other movelets of its mode, then it is probably not helpful in identifying that mode.

With a given time interval of length *T*, a GPS movelet of length *T* contains *T* data points, and an accelerometer movelet of length *T* contains 5T data points. The Euclidean distance incorporates five times as many points when calculating the distance between two accelerometer movelets. We scaled the distance calculation to account for the different lengths of the speed and acceleration movelets. For each validation movelet, we calculate the training movelet that results in the least squared distance and “match” these movelets. We count the number of times a training movelet is matched to a validation movelet of a given mode of transportation so that each training movelet has a corresponding count of the number of times it was matched to each mode of transportation. The pseudocode for matching movelets is described in Algorithm 2.

The range of the values for acceleration and speed varies widely. Calculating the distance between two sets of movelets without some sort of standardization would bias the results by overemphasizing the feature with the broader range. For this reason, all movelets of both features were scaled to the range [0,1]. This equalized the relative importance of the distance between both features.

### 3.3. Refining the Dictionary

**Figure 1:**
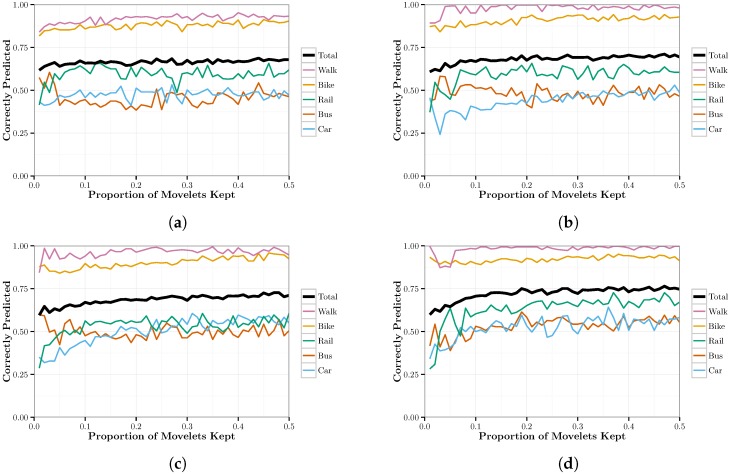
Graphs representing the proportion of correctly predicted movelets by mode of transportation as the proportion of movelets kept in a chapter is increased for movelets of a specified time length. (**a**) 30 s; (**b**) 60 s; (**c**) 90 s; (**d**) 120 s.

Next, we optimized the chapters so they only contained the most useful movelets for each mode of transportation. First, we assigned each mode of transportation a “chapter”, which is composed of all of the movelets for a given mode. Next, for each chapter, we ranked each movelet by the number of times it was used to identify a validation movelet. Then, we kept only the movelets that were in the top *n*-th percentile for some predefined percentage cutoff *n*. We tested all values of n∈{1,2,…,50}. A maximum value of 50 was sufficient to demonstrate the asymptotic behavior of the predictive accuracy of movelet dictionaries, as shown in Figure 1. Finally, we compared movelets across chapters and removed all ambiguous movelets. We defined a movelet to be ambiguous if it matched to multiple modes of transportation. The pseudocode for refining the training movelet dictionary is described in Algorithm 2.
**Algorithm 2** Pseudocode for Refining the Training Movelet DictionaryGiven movelet chapters $C_{train}$, $C_{val}$ and desired percentage cutoff *n*For each movelet Y in $C_{val}$:      For each movelet Z in $C_{train}$:           Calculate $d\left(Y,Z\right)={∥Y,Z∥}_{2}$ where $\left|\right|\cdot {\left|\right|}_{2}$ is the $\ell^{2}$ norm.      Match Y with the training movelet $Z^{\prime }$ that minimizes d(Y,Z)For each mode *m*:      Initialize chapter $C_{m}=\left\{\emptyset \right\}$      Include in $C_{m}$ all movelets $C_{train}$ that matched with any movelet of mode *m*      Let $\kappa_{m}$ represent the current number of movelets in $C_{m}$For each mode *m*:      Remove from $C_{m}$ any movelet that was matched with any class $m^{\prime }\neq m$      While the number of movelets in $C_{m}$ is greater than $\frac{100-n}{100}\kappa_{m}$:           Remove from $C_{m}$ the remaining movelet with the least number of matches

### 3.4. Predictions on New Data

Recall that H∈{30,60,90,120} represents the window size of the movelets. To predict the mode $\hat{M}\left(X\left(t\right)\right)$ based on a bivariate time series of new data, we computed the movelet X(t)=(A(t),S(t)) over the past *H* seconds and identified the chapter whose component movelets are closest to X(t), i.e.,

$$\hat{M}\left(X\left(t\right)\right)=\underset{m}{arg min}\min_{X_{j}\left(t^{\prime }\right)\in C_{m}}\frac{\left|\right|A_{j}\left(t^{\prime }\right),A\left(t\right){\left|\right|}_{2}}{f_{A}}+\frac{\left|\right|S_{j}\left(t^{\prime }\right),S\left(t\right){\left|\right|}_{2}}{f_{S}}$$

where $\left|\right|\cdot {\left|\right|}_{2}$ is the $\ell^{2}$ norm. Predictions are made for every movelet. Our results thus represent the accuracy of predicting individual movelets, rather than moments in time. By design, movelets are overlapping in time. Thus, each time, *t* will have multiple predictions. For real-time prediction, the most recent movelet mode prediction could be used. Alternatively, one could calculate the mode prediction of the past several movelets.

## 4. Feature-Based Methods

The movelets technique described above uses the “raw” GPS and accelerometer time series to identify unique signatures of modes of transportation. An alternative is to summarize characteristics, or features, of the time series and build models based on these features.

Formally, we define a feature F as a mapping from a stochastic processes to a scalar. Let $F_{S_{<}}\left(t,H\right)$ and $F_{A_{<}}\left(t,H\right)$ respectively denote the features calculated from the elements of $S_{<}\left(t\right)$ and $A_{<}\left(t\right)$ in the time interval [t,t−H]. For example, average speed over the past 30 s is given by:

$$F_{S_{<}}\left(t,30\right)=\frac{1}{30f_{S}}\sum_{j=1}^{30f_{S}}S\left(t-j/f_{S}\right)$$

In addition to calculating features from $S_{<}\left(t\right)$ and $A_{<}\left(t\right)$, we also calculated features from the iterated differences of speed and acceleration, i.e., by defining the stochastic processes:

$$\begin{array}{c} S_{\Delta }\left(t\right)=\left\{S\left(t-1/f_{S}\right)-S\left(t-2/f_{S}\right),S\left(t-2/f_{S}\right)-S\left(t-3/f_{S}\right),\ldots \right\}\\ A_{\Delta }\left(t\right)=\left\{A\left(t-1/f_{A}\right)-A\left(t-2/f_{A}\right),A\left(t-2/f_{A}\right)-A\left(t-3/f_{A}\right),\ldots \right\} \end{array}$$

and calculating features $F_{S_{\Delta }}$ and $F_{A_{\Delta }}$. As in the movelets models, we used random sampling to split the data into 60% training, 20% validation and 20% testing. Furthermore, as in the movelet models, we use Δ=5 to induce substantial overlap in our features. This allows for multiple predictions for any given time point and increases our sample size to better represent and identify a randomly selected segment of time.

We constructed features at 30-second intervals, for H= 30, 60, 90 and 120 s. Features calculated on speed, acceleration and their iterated differences were the mean, median, variance, minimum, maximum, interquartile range, 20th percentile and 80th percentile. Thus, we generated eight features for each of the four time windows for a total of 32 features for each of four defined stochastic processes. For the acceleration data only, we also calculated the autocorrelation, the sum of the first six real components of the Fourier transform of the sequence and the sum of the first six imaginary components of the Fourier transform of the sequence. This generated an additional 12 features from each of those three quantities calculated for each of the four time windows. Thus, we have 140 features, with N=8075 values for each feature.

We evaluated the performance of four techniques defined by combining two dimension reduction techniques, principal component analysis (PCA) and recursive feature elimination (RFE), and two popular machine learning techniques, *k*-nearest neighbors (*k*NN) and random forests (RF). In the following sections, we provide an overview of these techniques.

### 4.1. Feature-Based Classification Techniques

We considered two popular machine learning techniques for classification that could be trained offline and yield relatively simple prediction rules that are easily implemented on a smartphone: *k*-nearest neighbors (*k*NN) and random forests (RF). Both techniques are intuitively appealing for mode of transportation classification. *k*NN is appealing because different episodes of the same mode of transportation are likely to share many common feature characteristics. Random forest holds promise because thresholds for key features can strongly distinguish between modes (e.g., speed >40 mph rules out walking and biking).

#### 4.1.1. *k*-Nearest Neighbors

*k*NN is a supervised machine learning algorithm useful for classifying data into multiple classes. The main idea of *k*NN is to classify a data point based on the classification of the points in proximity, called the neighbors. It relies on a user-defined parameter *k*, which determines the number of neighbors to consider. We defined the *k*-nearest neighbors as the *k* training observations with the least Euclidean distance to the point being classified. The class of each of the *k*-nearest neighbors was counted as votes, and the majority vote determined the class of the testing data. We used the class package in R [22]. Ties were broken at random.

We used leave-one-out cross-validation on the training data to determine the optimal value of *k*. Leave-one-out cross-validation is equivalent to *N*-fold cross-validation where *N* is the total number of points in the data. (N−1) data points were used to predict the class of the last data point. This process was repeated until every point had been left out. The true error rate was estimated to be the average error rate of the *N* trials. We cross-validated all values from k=1 to k=100. The value of *k* that resulted in the most accurate cross-validated prediction was used in generating the final model to predict the test data. While straightforward to implement, *k*NN does pose some computational burden, as the nearest neighbor(s) of any input data must be computed.

#### 4.1.2. Random Forests

Random forests use “ensemble learning”, a method hat generates many classifiers and then aggregates their results to output predictions. Each decision tree was constructed using a random bootstrap sample of the training data. Input observations were classified by every tree in a random forest. The plurality vote determined the final classification to prevent overfitting [23]. A majority of votes from the predictions of single trees is known to give higher accuracy than the use of a single tree, which makes random forests an optimal choice over the use of only one decision tree [24].

There are several measures available to determine which variable ‘best’ splits the data at each node within the component trees of a random forest. We used the Gini impurity index, which is defined for a node *A* as follows:

(1) $$I\left(A\right)=\sum_{i=1}^{C}p_{iA}\left(1-p_{iA}\right)$$

where the sum is over the *C* classes and $p_{iA}$ is the proportion of items in class *i* at node *A*. The Gini impurity of a node measures the probability that a randomly chosen item from the node would be incorrectly labeled when randomly assigned a label in accordance with the observed distribution of labels in the node. The feature that best splits the data is that which minimizes the Gini index.

We built random forests from the training data using the randomForest package in R [25]. We used 500 recursive partition trees in each random forest model and considered the square root of the total number of features at each split. Note that obtaining predictions from a random forest model fitted offline poses a particularly low computational burden on the phone, as only a sequence of inequalities on the features must be evaluated. Additionally, cross-validation is not necessary in order to get an unbiased estimate of out-of-sample error [23].

### 4.2. Dimension Reduction for Speed and Acceleration Features

To achieve near-real time prediction of the mode of transportation, the smartphone would have to calculate the aforementioned 140 features every 30–120 s, which is computationally intensive. In our tests, feature computation was a substantial contributor to battery drain, so we investigated whether it was possible to achieve good prediction accuracy with a smaller number of features. We considered two methods for reducing the dimension of the feature space by applying either feature extraction via PCA or feature selection via RFE.

#### 4.2.1. Principal Component Analysis

Principal component analysis (PCA) is a linear transformation of a feature set often used as a dimension reduction algorithm. It transforms features of the data into orthogonal vectors called principal components. The idea is to represent high-dimensional data on a low-dimensional subspace while maintaining most of the variation in the data. The principal components are the orthogonal vectors that span this subspace. We projected each observation in our dataset onto this orthogonal space and recorded the coordinates. We then used classification techniques to classify the data based on these coordinates.

For PCA, we projected each observation onto its principal components and then used classification techniques on these new coordinates. Since PCA is sensitive to feature scaling, the first step in these models was to standardize the features. This involved centering each feature to have a mean of zero by subtracting the mean and scaling each feature to have a variance of one by dividing by the standard deviation. This ensured that the variance of one feature does not outweigh the variance of another. PCA calculations were carried out using the princomp() function in R.

#### 4.2.2. Recursive Feature Elimination

RFE is an algorithm used to determine the top untransformed features of a dataset. It is a form of backward elimination, iteratively removing features until an optimal number is found. First, the algorithm partitions the data into training and testing sets via resampling. The algorithm creates a classification model using the full set of *n* predictors on the training set and uses this model to predict the test set. Each predictor is ranked according to its contribution to improving the accuracy of the model. Next, it creates a subset composed of the top *j* features for all integer values of *j* such that j≤n. Each subset is used to create another classification model employing only the top *j* features, and this model is adopted to predict the test set. This entire process is repeated until the desired number of folds for cross-validation is reached. After testing all values of *j*, the algorithm identifies the value with the highest average prediction accuracy. Label this value *k* (the optimal number of features) and return the top *k* features. In our implementation, we used 10-fold cross-validation and the random forest algorithm to rank the top features. We also compared all possible values of *j*, rather than keeping only the optimum. We used the implementation from the caret package in R [26].

### 4.3. Model Efficiency Metric

We aimed to evaluate not only the absolute accuracy of our models, but also the accuracy relative to the percent of variance of the original data retained. We created the following metric, which we call the model efficiency metric (MEM), to capture this tradeoff:

(2) ModelEfficiencyMetric=(ClassificationAccuracy)×(1−%VarianceRetained)

where classification accuracy is the percent of correctly classified observations. In other words, we weighted the model classification accuracy by the percent of variance removed from the training data used to construct the model. The percent of variance removed from the training data serves as a surrogate for how much processing a smartphone would have to perform on raw sensor data in order to calculate the necessary features to implement the model. With this metric, a given model might be considered superior to another if it has a slightly lower accuracy, but a significantly reduced percentage of variance retained.

When selecting the optimal model from a group of models, we wanted to maximize the MEM, but we did not want to use a model that has a classification accuracy less than 90 percent of the maximum classification accuracy in the group of comparison. Thus, we imposed the constraint of Equation (3) on our models to identify an optimal model and balance the goals of dimension reduction and model accuracy. Letting $M=\{M_{1},M_{2},\ldots ,M_{n}\}$ be a set of *n* models with estimated classification accuracy rates $\left\{\alpha_{1},\ldots ,\alpha_{n}\right\}$ and defining $\alpha_{max}=\max\left\{\alpha_{1},\alpha_{2},\ldots ,\alpha_{n}\right\}$, we define the optimal model $M_{*}$ from the set M as:

(3) $$\begin{array}{ccc} M_{*}=\; & \underset{i}{\mathrm{maximize}} & MEM\left(M_{i}\right)=\alpha_{i}\left(1-\gamma_{i}\right)\\ & \mathrm{subject}\;\mathrm{to} & \alpha_{i}\geq 0.9\;\alpha_{\max},\;\;i=1,\ldots ,n \end{array}$$

## 5. Results

### 5.1. Movelets

We calculated movelets for four time intervals: 30, 60, 90 and 120 s. Movelets shorter than 30 s do not contain enough information to classify accurately. Movelets longer than 120 s are impractical for near-real-time classification and limit the size our dataset. Figure 1 represents the proportion of correctly predicted movelets by mode of transportation as the proportion of movelets increases. This shows how much an additional unit in the chapter helps in mode identification. In general, Figure 1 shows that the accuracy, or recall, of the predictions increases as we increase the proportion of movelets used in prediction.

**Figure 2:**
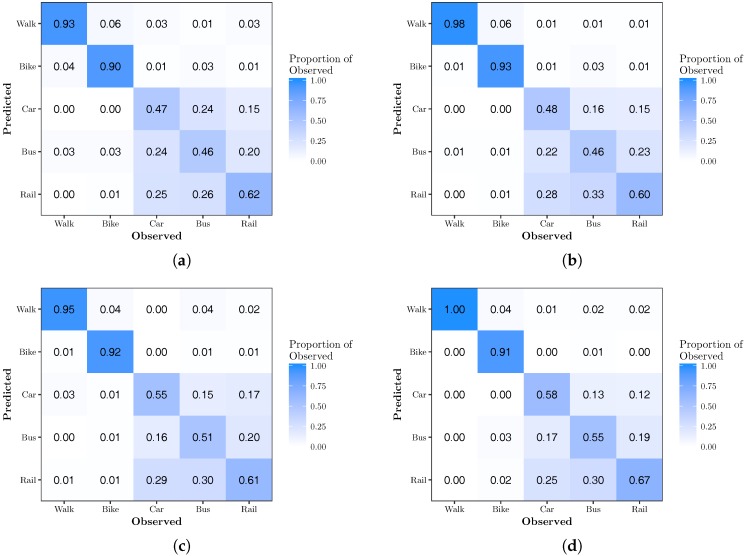
Matrices representing the proportion of observed modes predicted in each category using movelets of a specified time length. (**a**) 30 s; (**b**) 60 s; (**c**) 90 s; (**d**) 120 s.

Figure 2 shows the percentage correctly predicted by mode of transportation using the top 50% of movelets from the validation. These results contain a significant amount of misclassification error, especially between the motorized forms of transportation. Walking and biking maintained classification accuracies above 90%. Car, bus and rail accuracies varied from 46–67%.

### 5.2. kNN with PCA (kNN-PCA)

We fit 31 *k*NN models on training data that were processed using PCA. We chose 31 to retain 95% of the variance of the original data. Each model was trained with a different number of the top principal components, ranging from 1–31. We refer to these models as the PCA-*k*NN models. PCA-*k*NN{*i*} represents the PCA-*k*NN model trained with the top *i* principal components of the training data.

**Figure 3:**
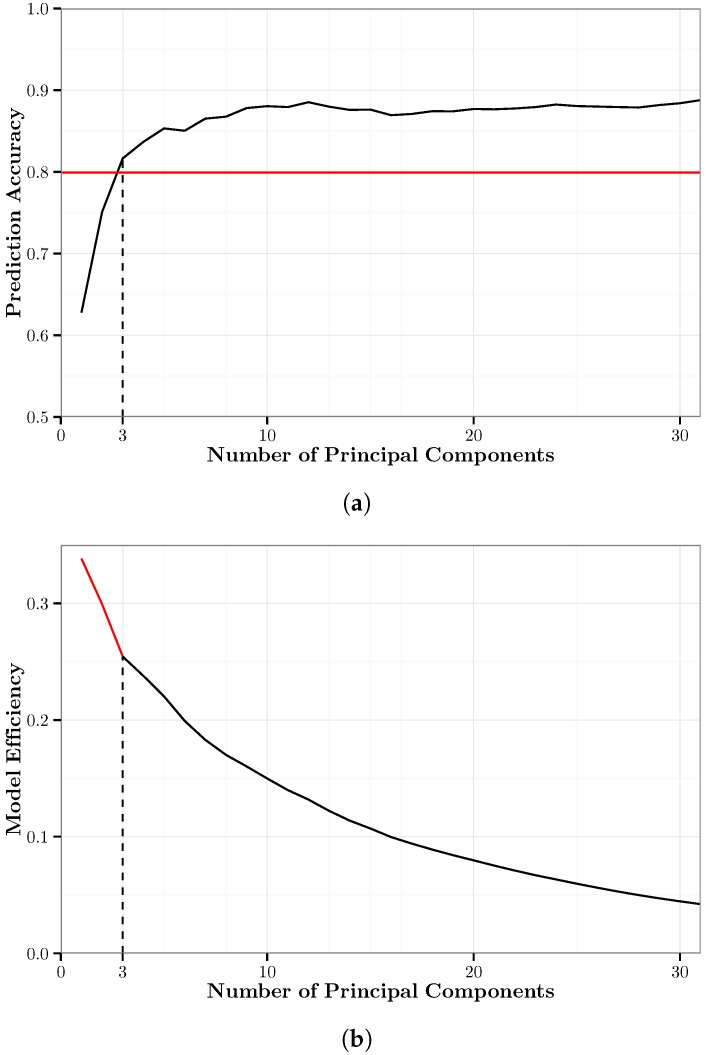
A graphical summary of the PCA-*k*NN models. (**a**) Ten-fold cross-validation accuracy of the PCA-*k*NN models. The solid black line represents the prediction accuracy; the solid red line represents the minimum cut-off accuracy for the optimal model; and the dashed black line represents the minimum number of principal components necessary to attain the cut-off accuracy. (**b**) Model efficiency metric of the PCA-*k*NN models. The red portion represents models that do not meet the cut-off accuracy; the black portion represents models that do meet the cut-off accuracy.

Figure 3a shows the overall accuracy of the PCA-*k*NN models and the minimum cut-off accuracy as defined in the constraint of Equation (3). Figure 3b shows the MEM (Equation (2)) of the PCA-*k*NN models. It shows that PCA-*k*NN{3} is the optimal PCA-*k*NN model based on this metric. PCA-*k*NN{31} is the highest accuracy PCA-*k*NN model with an accuracy of 88.8% using 95.3% of the variance of the original data. PCA-*k*NN{3} has an overall accuracy of 83.5% using only 68.8% of the variance of the original data. Compared to PCA-*k*NN{31}, this is a decrease in accuracy of only 5.3 percentage points using 26.5 percentage points less of the original variance.

**Figure 4:**
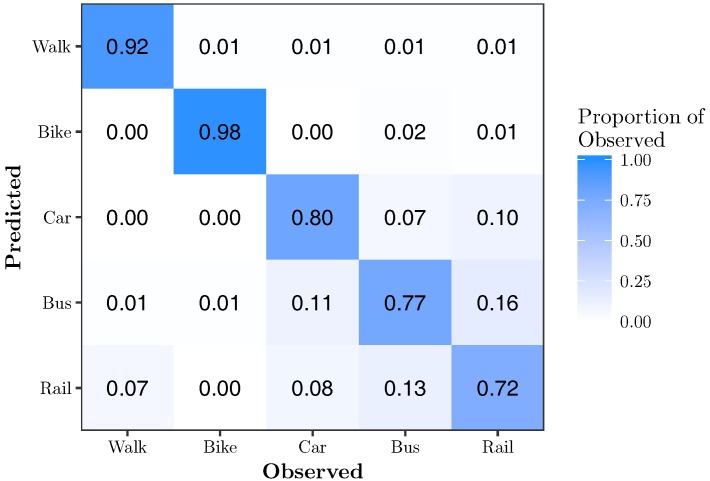
Confusion matrix for PCA-*k*NN{3}. This model has an overall accuracy of 83.5% using 68.8% of the original feature variation.

Figure 4 shows the confusion matrix for PCA-*k*NN{3}. For these models, three principal components are sufficient to very accurately classify walking and biking, with 92% and 98% accuracy, respectively. There is still moderate confusion between car, bus, and rail, which are classified with 80%, 77% and 72% accuracy, respectively.

### 5.3. kNN with RFE (kNN-RFE)

We fit 70 *k*NN models on training data that were processed using RFE. We chose 70 in order to retain half of the original 140 features. We refer to these models as the RFE-*k*NN models. RFE-*k*NN{*i*} represents the RFE-*k*NN model trained with the top *i* features of the training data chosen by RFE. For all RFE-*k*NN models, the optimal value of *k* was determined to be one.

**Figure 5:**
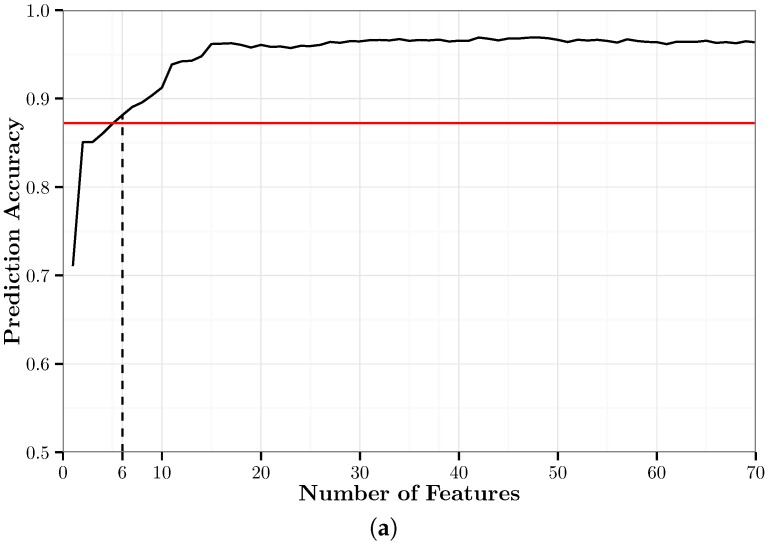

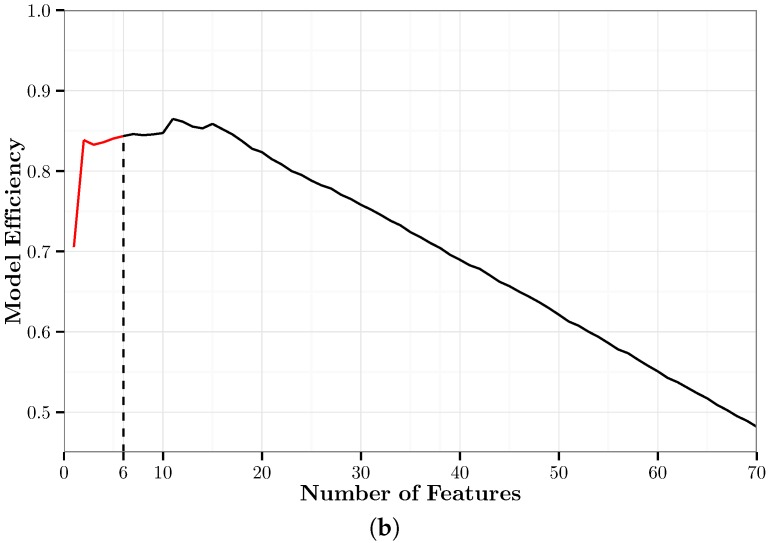
A graphical summary of the recursive feature elimination (RFE)-*k*NN models. (**a**) Ten-fold cross-validation accuracy of the RFE-*k*NN models. The solid black line represents the prediction accuracy; the solid red line represents the minimum cut-off accuracy for the optimal model; and the dashed black line represents the minimum number of features necessary to attain the cut-off accuracy. (**b**) Model efficiency metric of the RFE-*k*NN models. The red portion represents models that do not meet the cut-off accuracy; the black portion represents models that do meet the cut-off accuracy.

Figure 5a shows the overall accuracy of the RFE-*k*NN models and the minimum cut-off accuracy as defined in the constraint of Equation (3). Figure 5b shows the MEM (Equation (2)) of the RFE-*k*NN models. It shows that RFE-*k*NN{11} is the optimal RFE-*k*NN model based on this metric. RFE-*k*NN{48} is the highest accuracy RFE-*k*NN model with an accuracy of 96.9% using 34.3% of the features. RFE-*k*NN{11} has an overall accuracy of 93.7% using only 7.9% of features. Compared to RFE-*k*NN{48}, this is a decrease in accuracy of only 3.2 percentage points using 26.4 percentage points less of the features.

**Figure 6:**
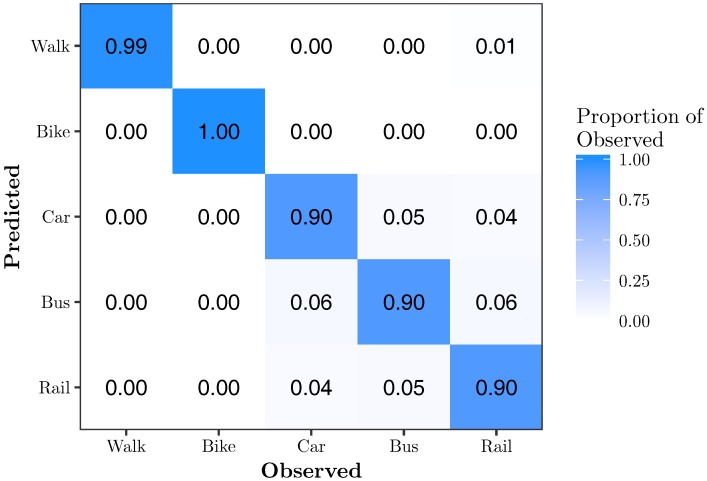
Confusion matrix for RFE-*k*NN{11}. This model has an overall accuracy of 93.7% using 7.9% of the features.

Figure 6 shows the confusion matrix for RFE-*k*NN{11}. It shows that 11 features is sufficient to classify all modes of transportation with very high accuracy. Walk, bike, car, bus, and rail are classified with 99%, 100%, 90%, 90% and 90% accuracy, respectively. There is still minor confusion between the motorized modes of transportation, while walking and biking are classified almost perfectly.

### 5.4. Random Forests with PCA (RF-PCA)

We generated 31 random forest models trained with data that were processed with PCA. Each model was trained with a different number of the top principal components, ranging from 1–31. We refer to these models as the PCA-RF models. PCA-RF{*i*} represents the PCA-RF model trained with *i* principal components of the training data. 

**Figure 7:**
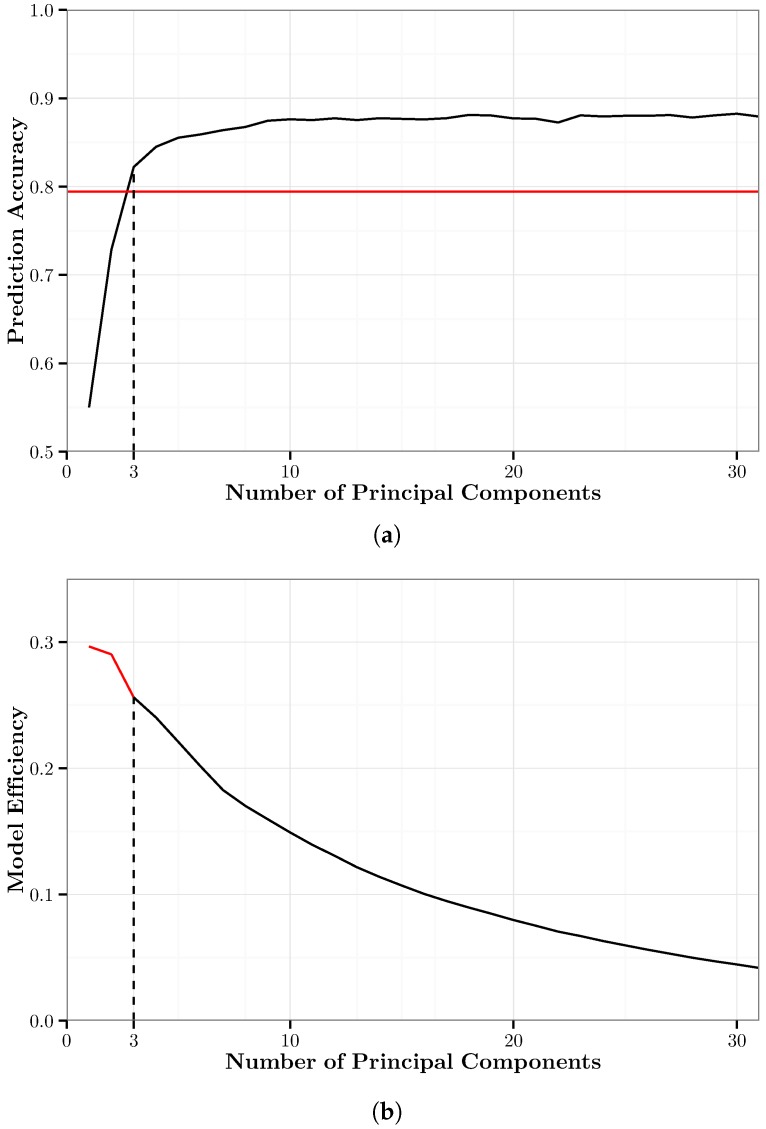
A graphical summary of the PCA-RF models. (**a**) Ten-fold cross-validation accuracy of the PCA-RF models. The solid black line represents the prediction accuracy; the solid red line represents the minimum cut-off accuracy for the optimal model; and the dashed black line represents the minimum number of principal components necessary to attain the cut-off accuracy. (**b**) Model efficiency metric of the PCA-RF models. The red portion represents models that do not meet the cut-off accuracy; the black portion represents models that do meet the cut-off accuracy.

We used 10-fold cross-validation to train each of the PCA-RF models. Figure 7a shows the overall accuracy of the PCA-RF models and the minimum cut-off accuracy as defined in the constraint of Equation (3). Figure 7b shows the MEM (Equation (2)) of the PCA-RF models. It shows that PCA-RF{3} is the optimal PCA-RF model based on this metric. This is the same amount of principal components used in the optimal PCA-*k*NN model. PCA-RF{30} is the highest accuracy PCA-RF model with an accuracy of 88.2% using 95.0% of the variance of the original data. PCA-RF{3} has an overall accuracy of 82.0% using only 68.8% of the variance of the original data. Compared to PCA-RF{30}, this is a decrease in accuracy of only 6.2 percentage points using 26.2 percentage points less of the original variance.

**Figure 8:**
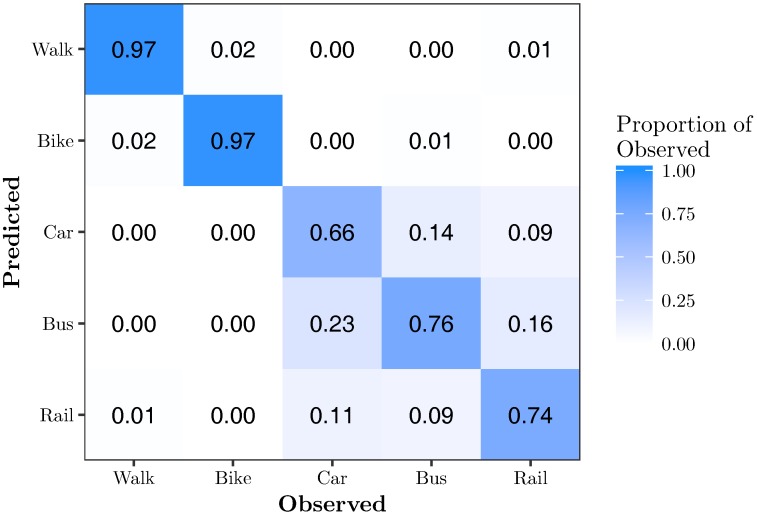
Confusion matrix for the optimal PCA-RF model, PCA-RF{3}. This model has an overall accuracy of 82.0% using 68.8% of the original feature variation.

Figure 8 shows the confusion matrix for PCA-RF{3}. Similar to PCA-*k*NN{3}, three principal components are sufficient to very accurately classify walking and biking, each with 97% accuracy. There is still significant confusion between car, bus and rail, which are classified with 66%, 76% and 74% accuracy, respectively.

### 5.5. Random Forests with RFE

We generated 70 random forest models trained with data that were processed with RFE. We refer to these models as the RFE-RF models. RFE-RF{*i*} represents the RFE-RF model trained with the top *i* features of the training data chosen by RFE. 

**Figure 9:**
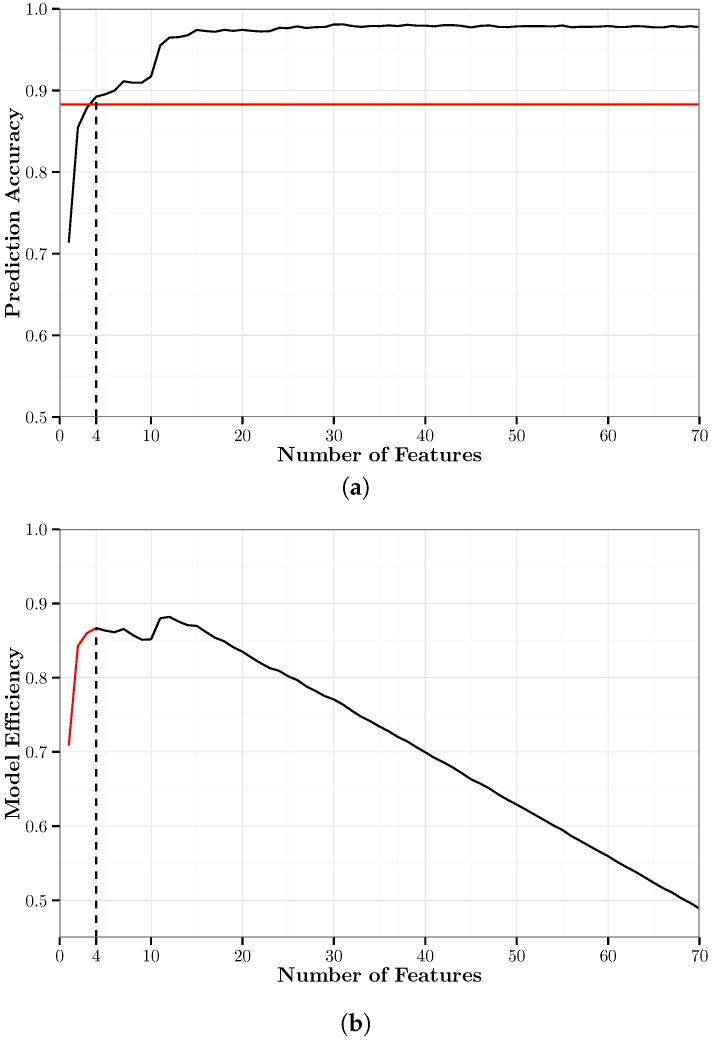
A graphical summary of the RFE-RF models. (**a**) Ten-fold cross-validation accuracy of the RFE-RF models. The solid black line represents the prediction accuracy; the solid red line represents the minimum cut-off accuracy for the optimal model; and the dashed black line represents the minimum number of principal components necessary to attain the cut-off accuracy. (**b**) Model efficiency metric of the RFE-RF models. The red portion represents models that do not meet the cut-off accuracy; the black portion represents models that do meet the cut-off accuracy.

We use 10-fold cross-validation to train each of the RFE-RF models. Figure 9a shows the overall accuracy of the RFE-RF models and the minimum cut-off accuracy as defined in the constraint of Equation (3). Figure 9b shows the MEM (Equation (2)) of the PCA-RF models. It shows that RFE-RF{12} is the optimal RFE-RF model. This uses one more feature than the optimal RFE-*k*NN{11} model. RFE-RF{31} is the highest accuracy RFE-RF model with an accuracy of 98.1% using 22.1% of the features. RFE-RF{12} has an overall accuracy of 96.8% using only 8.6% of the features. Compared to RFE-RF{31}, this is a decrease in accuracy of only 1.3 percentage points using 13.5 percentage points less of the original variance. 

**Figure 10:**
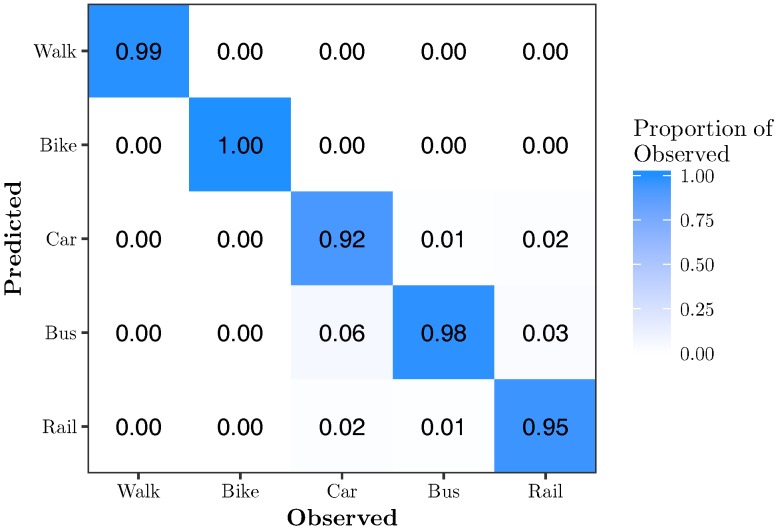
Confusion matrix for RFE-RF{12}. This model has an overall accuracy of 96.8% using 8.6% of the features.

Figure 10 shows the confusion matrix for RFE-RF{12}. It shows that 12 features are sufficient to classify all modes of transportation with very high accuracy. Walk, bike, car, bus and rail are classified with 99%, 100%, 92%, 98% and 95% accuracy, respectively. 

**Figure 11:**
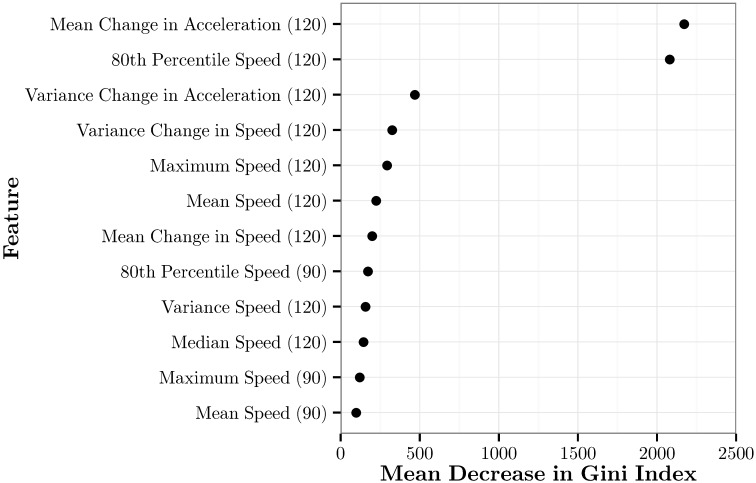
The mean decrease in the Gini index for each of the top 12 features in RFE-RF{12}.

The feature importance of the top 12 features in RFE-RF{12} based on the mean decrease in the Gini index is shown in Figure 11. Two features greatly outperform the rest. These are mean change in acceleration over the past 120 s and the 80th percentile of speed in the past 120 s. Ten of the twelve most important features are speed features. Interestingly, the two acceleration features are two of the top three features overall. The top seven features are all 120-s features. Of the remaining five features, two are 120-s features, and three are 90-s features. Additionally, for all of the 90-s features that are there, the 120-s equivalent is also included. No 60-s or 30-s features were used.

## 6. Discussion

In our examinations, we found that movelets performed poorly in predicting the mode of travel. One possible explanation is that our movelet dictionaries are simply too small. We observed an overall trend of increasing classification accuracy as the number of movelets in a chapter is increased. However, this may be offset by the rapidly diminishing returns in accuracy of additional movelets beyond a certain threshold, as Figure 1 strongly suggests an asymptotic limit to the predictive accuracy of the movelet models. Additionally, movelet models are the only models in this paper for which the computational cost of implementation scales with the size of the training data due to the distance calculations. This, combined with the diminishing returns to predictive accuracy as the number of movelets in a dictionary is increased, suggests that increasing dictionary size would not be worthwhile. It is also possible that we did not generate movelets on the appropriate features. It is reasonable to assume that accuracy would improve if we were able to generate movelets separately for all three dimensions of the tri-axial accelerometer. Again, this would greatly increase the computational cost. Additionally, other classification algorithms were able to achieve high accuracy without utilizing all three dimensions. 

Another finding is that, used in conjunction with random forests or *k*-nearest neighbors, RFE substantially outperforms PCA. Methods incorporating RFE were both more accurate and retained much less variation. We hypothesize that the relatively poor performance of our PCA models is due to the linearity of the PCA algorithm. It is possible that the feature space for predicting mode of transportation would be better transformed using a nonlinear technique. 

Table 1 shows the percent correctly, by mode of transportation, using the optimal version of each of the five methods implemented in this paper. Overall, our methodology suggests a random forest classification algorithm on features selected using RFE. RFE-RF{12} requires only 12 features and achieved an accuracy of 96.8%. Even the three different motorized modes of transportation were each classified with at least 92% accuracy. Further, this model is relatively computationally efficient, requiring neither cross-validation nor a transformation of the feature space. It also allows for an easy examination of the features that are most useful for mode classification. 

Our models were developed assuming no information about a user’s frequency of different trip types in an attempt to make them as generalizable as possible. In practice, one might enter an effective weight on the various modes of transportation. For example, a user might inform the model that he/she seldom uses a bus. Such information would likely improve the predictive accuracy of any model for that user. 

The fact that most of the key features are speed features suggests that speed is more useful than acceleration in distinguishing between modes of transportation, but some feature of acceleration is still required. While most decisions in the random forest may split on a speed feature, these splits would not be as effective without also splitting on an acceleration feature. Hence, acceleration features may be thought of as somewhat of a catalyst for the efficacy of speed features. Moreover, features that were generated over a longer time interval (90–120 s) outperformed those generated over shorter time intervals (30–60 s). 

One limitation of our approach is that our use of variance retained as an efficiency metric may bias us in favor of RFE. PCA is designed to capture as much variance as possible in as few components as possible. Thus, no matter how few components we used, we were likely to retain at least a moderate percentage of the original variance. An alternative approach could compare the number of principal components used in PCA models to the number of features retained in RFE models. We argue, however, that it is necessary to compare PCA models to RFE models by their variance retained since, without knowing variance retained, we would not be able to generate principal components for a model. When attempting to implement a PCA model on new data, we would need to construct the principal components of the new data using many features. It would therefore bias our results in favor of PCA if we were to compare reduction efficiency based only on the number of principal components used. It may be possible to generate a classification model using PCA on less features while maintaining most of the classification accuracy by combining PCA with a feature selection technique, but we did not attempt this. 

A possible critique of our method is that we only classified trips that were known to be of a single mode of transportation. While it is true that this might diminish our ability to predict transitions between modes, this is not a goal of the current paper. Instead, we are interested in how accurately we can distinguish between segments of time that include single and diverse modes. An interesting extension of our work might include a method to quantify the confidence of classification and to use this confidence in identifying periods of transition.

A possible improvement to our classifier would be the incorporation of more training data. While our data were sufficient to achieve a promising predictive accuracy, more data coming from a broader variety of locations could help improve classification accuracy, especially between motorized transportation. Another possible extension of our work would be to experiment with different dimension reduction techniques and classification algorithms. *k*NN and RF algorithms both proved to be very effective classifiers, but other approaches such as support vector machines or naive Bayes could also be considered. Additionally, we used relatively simple summary statistics for the bivariate speed-acceleration time series to derive features. Our initial investigations showed that signal processing-based features (e.g., Fourier coefficients) did not improve predictions, but more research is needed to confirm this preliminary finding. 

## Figures and Tables

**Table 1:** Percent correctly predicted, by mode of transportation, using the optimal version of each method.

Mode of Transportation	Movelets	*k*-Nearest Neighbor		Random Forest	
		PCA-*k*NN	RFE-*k*NN	PCA-RF	RFE-RF
Walk	100	92	99	97	99
Bike	91	98	100	97	100
Car	58	80	90	66	92
Bus	55	77	90	76	98
Rail	67	72	90	74	95
Total	74	84	94	82	97
